# Diammonium 1,1′,3,3′-tetra­methyl-2,2′,4,4′,6,6′-hexa­oxoperhydro-5,5′-bipyrimidine-5,5′-diide monohydrate

**DOI:** 10.1107/S1600536809039968

**Published:** 2009-10-07

**Authors:** Hoong-Kun Fun, Jia Hao Goh, B. Palakshi Reddy, V. Vijayakumar, S. Sarveswari

**Affiliations:** a X-ray Crystallography Unit, School of Physics, Universiti Sains Malaysia, 11800 USM, Penang, Malaysia; b Organic Chemistry Division, School of Science, VIT University, Vellore 632 014, India

## Abstract

In the title hydrated salt, 2NH_4_^+^·C_12_H_12_N_4_O_6_^2−^·H_2_O, the two hexa­hydro­pyrimidine rings in the dianion are inclined to one another at a dihedral angle of 62.76 (5)°. In the crystal structure, the anions and water mol­ecules are linked into sheets parallel to the *bc* plane by inter­molecular O—H⋯O hydrogen bonds and sustained by C—H⋯O contacts. The linking of the anions and water mol­ecules with the cations by N—H⋯O hydrogen bonds creates a three-dimensional extended network. The crystal structure is further stabilized by very weak C—H⋯π inter­actions.

## Related literature

For general background to and applications of barbituric acid derivatives, see: Negwer (2001 ▶). For related structures, see: Rezende *et al.* (2005 ▶); da Silva *et al.* (2005 ▶). For the stability of the temperature controller used for the data collection, see: Cosier & Glazer (1986 ▶). 
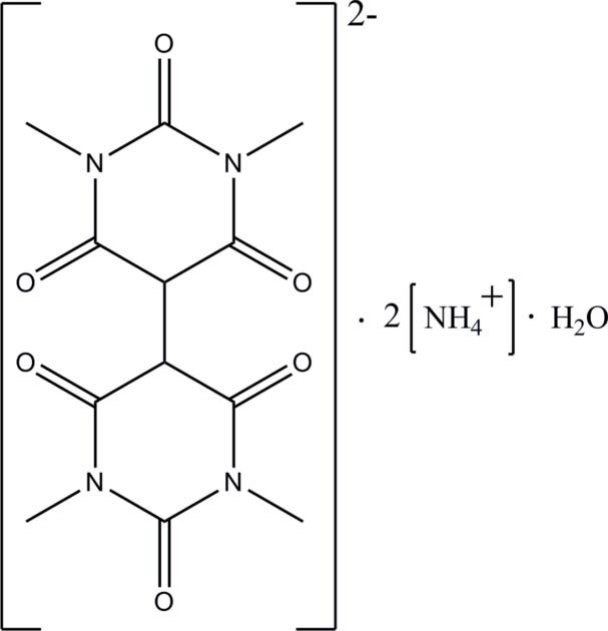


## Experimental

### 

#### Crystal data

2NH_4_^+^·C_12_H_12_N_4_O_6_^2−^·H_2_O
*M* *_r_* = 362.36
Monoclinic, 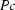

*a* = 8.5345 (1) Å
*b* = 12.1579 (2) Å
*c* = 7.7482 (1) Å
β = 100.595 (1)°
*V* = 790.26 (2) Å^3^
*Z* = 2
Mo *K*α radiation
μ = 0.13 mm^−1^
*T* = 100 K
0.46 × 0.24 × 0.20 mm

#### Data collection

Bruker SMART APEXII CCD area-detector diffractometer
Absorption correction: multi-scan (**SADABS**; Bruker, 2005 ▶) *T*_min_ = 0.945, *T*_max_ = 0.975
15091 measured reflections
3387 independent reflections
3237 reflections with *I* > 2σ(*I*)
*R*_int_ = 0.026

#### Refinement

*R*[*F* ^2^ > 2σ(*F* ^2^)] = 0.029
*wR*(*F* ^2^) = 0.083
*S* = 1.05
3387 reflections
270 parameters
2 restraints
H atoms treated by a mixture of independent and constrained refinement
Δρ_max_ = 0.41 e Å^−3^
Δρ_min_ = −0.22 e Å^−3^

### 

Data collection: *APEX2* (Bruker, 2005 ▶); cell refinement: *SAINT* (Bruker, 2005 ▶); data reduction: *SAINT*; program(s) used to solve structure: *SHELXTL* (Sheldrick, 2008 ▶); program(s) used to refine structure: *SHELXTL*; molecular graphics: *SHELXTL*; software used to prepare material for publication: *SHELXTL* and *PLATON* (Spek, 2009 ▶).

## Supplementary Material

Crystal structure: contains datablocks global, I. DOI: 10.1107/S1600536809039968/tk2548sup1.cif

Structure factors: contains datablocks I. DOI: 10.1107/S1600536809039968/tk2548Isup2.hkl

Additional supplementary materials:  crystallographic information; 3D view; checkCIF report

## Figures and Tables

**Table 1:** Hydrogen-bond geometry (Å, °)

*D*—H⋯*A*	*D*—H	H⋯*A*	*D*⋯*A*	*D*—H⋯*A*
N5—H1*N*5⋯O1^i^	0.87 (2)	2.04 (2)	2.7629 (14)	141 (2)
N5—H1*N*5⋯O6^i^	0.87 (2)	2.52 (2)	3.1697 (14)	132.9 (19)
N5—H2*N*5⋯O1^ii^	0.85 (2)	1.97 (2)	2.8116 (14)	173 (2)
N5—H3*N*5⋯O5	0.94 (3)	1.90 (3)	2.8312 (14)	176.1 (19)
N5—H4*N*5⋯O6^iii^	0.89 (2)	1.90 (2)	2.7819 (14)	173 (2)
N6—H1*N*6⋯O1*W*	0.88 (2)	2.10 (2)	2.9126 (15)	152 (2)
N6—H2*N*6⋯O2^iv^	0.93 (3)	2.03 (2)	2.9074 (15)	157.8 (19)
N6—H3*N*6⋯O4^v^	0.84 (2)	1.95 (2)	2.7665 (14)	164 (2)
N6—H4*N*6⋯O3	0.88 (2)	1.92 (2)	2.7597 (14)	158 (2)
O1*W*—H1*W*1⋯O3^v^	0.84 (3)	1.96 (2)	2.7602 (13)	158 (2)
O1*W*—H2*W*1⋯O4^vi^	0.78 (3)	2.01 (3)	2.7563 (14)	161 (3)
C9—H9*A*⋯O1*W*^vii^	0.96	2.51	3.3658 (17)	148
C10—H10*C*⋯*Cg*1^v^	0.96	2.96	3.8822 (15)	162

Symmetry codes: (i) 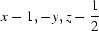; (ii) 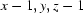; (iii) 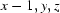; (iv) 
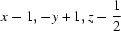
; (v) 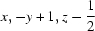; (vi) 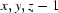; (vii) 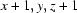. *Cg*1 is the centroid the the C1/N1/C2/N2/C3/C4 ring.

**Fig. 1.**
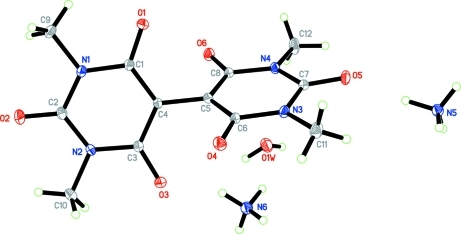
The molecular structure of (I), showing 50% probability displacement ellipsoids for non-H atoms and the atom-numbering scheme.

**Fig. 2.**
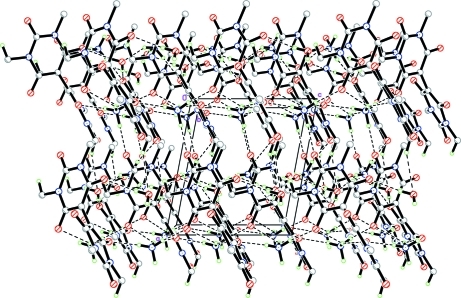
The crystal structure of (I) viewed along the b axis, showing the three-dimensional network. H atoms not involved in intermolecular interactions (dashed lines) have been omitted for clarity.

## supplementary crystallographic information

### Comment

Barbituric acid derivatives show high hypnotic and sedative activity (Negwer, 2001).

The asymmetric unit of the title hydrated salt (I, Fig. 1) contains two ammonium cations, a 1,1',3,3'-tetramethyl-2,2',4,4',6,6'-hexaoxooctahydro-1*H*, 1'*H*-5,5'-bipyrimidine-5,5'-diide dianion and a water molecule. Two protons transfer from the C4 and C5 atoms to the ammonia molecules resulted in the formation of salts. The dianion is built up from one dimethylbarbiturate anion connected to the other one through the C*_sp_*^3^—C*_sp_*^3^ (C4—C5) bond. The two hexahydropyrimidine rings are essentially planar, with maximum deviations of 0.036 (1) Å for atom C1 and 0.018 (1) Å for atom N3, respectively, for rings with atom sequence C1/N1/C2/N2/C3/C4 and C5/C6/N3/C7/N4/C8. These two rings are inclined to one another at a dihedral angle of 62.76 (5)°. The bond lengths and angles are comparable to those found in related structures (Rezende *et al.*, 2005; da Silva *et al.*, 2005).

The crystal structure of (I) is mainly stabilized by a network of N—H···O, and O—H···O hydrogen bonds as well as C—H···O and C—H···π contacts. Each ammonium H-atom participates in intermolecular hydrogen bonds. In the crystal structure (Fig. 2), the anions and water molecules are linked into sheets parallel to the *bc* plane by O—H···O hydrogen bonds and sustained by C—H···O contacts  (Table 1). The ammonium cations act as bridges between the anions and water molecules *via* N—H···O hydrogen bonds (Table 1) to create a three-dimensional extended network. The crystal structure is further stabilized by weak intermolecular C10—H10C···*Cg*1 interactions (Table 1).

### Experimental

A solution of 1,3-dimethylbarbituric acid was refluxed in acetonitrile at 363 K for 2 h (monitored by TLC). After completion of the reaction, excess of solvent was distilled off. The solid product obtained was washed with mixture of ether and acetone, and dried. The purity of the crude product was checked through TLC and recrystallized using chloroform and benzene mixture. *M.p.* 515–517 K.

### Refinement

The H-atoms bound to atoms N5, N6 and O1W were located from the difference Fourier map and allowed to refine freely. The other H-atoms were placed in calculated positions, with C—H = 0.96 Å, *U*_iso_ = 1.5*U*_eq_(C). Rotating models were used for the methyl groups. In the absence of significant anomalous dispersion, 2042 Friedel pairs were merged for the final refinement.

### Crystal data

**Table:**

2NH_4_^+^·C_12_H_12_N_4_O_6_^2^^−^·H_2_O	*F*(000) = 384
*M**_r_* = 362.36	*D*_x_ = 1.523 Mg m^−^^3^
Monoclinic, *P**c*	Mo *K*α radiation, λ = 0.71073 Å
Hall symbol: P -2yc	Cell parameters from 9975 reflections
*a* = 8.5345 (1) Å	θ = 3.0–34.6°
*b* = 12.1579 (2) Å	µ = 0.13 mm^−^^1^
*c* = 7.7482 (1) Å	*T* = 100 K
β = 100.595 (1)°	Block, yellow
*V* = 790.26 (2) Å^3^	0.46 × 0.24 × 0.20 mm
*Z* = 2	

### Data collection

**Table:**

Bruker SMART APEXII CCD area-detector diffractometer	3387 independent reflections
Radiation source: fine-focus sealed tube	3237 reflections with *I* > 2σ(*I*)
graphite	*R*_int_ = 0.026
φ and ω scans	θ_max_ = 34.7°, θ_min_ = 2.4°
Absorption correction: multi-scan (*SADABS*; Bruker, 2005)	*h* = −13→13
*T*_min_ = 0.945, *T*_max_ = 0.975	*k* = −19→19
15091 measured reflections	*l* = −12→12

### Refinement

**Table:**

Refinement on *F*^2^	Primary atom site location: structure-invariant direct methods
Least-squares matrix: full	Secondary atom site location: difference Fourier map
*R*[*F*^2^ > 2σ(*F*^2^)] = 0.029	Hydrogen site location: inferred from neighbouring sites
*wR*(*F*^2^) = 0.083	H atoms treated by a mixture of independent and constrained refinement
*S* = 1.05	*w* = 1/[σ^2^(*F*_o_^2^) + (0.0579*P*)^2^ + 0.0092*P*] where *P* = (*F*_o_^2^ + 2*F*_c_^2^)/3
3387 reflections	(Δ/σ)_max_ < 0.001
270 parameters	Δρ_max_ = 0.41 e Å^−^^3^
2 restraints	Δρ_min_ = −0.22 e Å^−^^3^

### Special details

**Table:**

Experimental. The crystal was placed in the cold stream of an Oxford Cyrosystems Cobra open-flow nitrogen cryostat (Cosier & Glazer, 1986) operating at 100.0 (1)K.
Geometry. All esds (except the esd in the dihedral angle between two l.s. planes) are estimated using the full covariance matrix. The cell esds are taken into account individually in the estimation of esds in distances, angles and torsion angles; correlations between esds in cell parameters are only used when they are defined by crystal symmetry. An approximate (isotropic) treatment of cell esds is used for estimating esds involving l.s. planes.
Refinement. Refinement of F^2^ against ALL reflections. The weighted R-factor wR and goodness of fit S are based on F^2^, conventional R-factors R are based on F, with F set to zero for negative F^2^. The threshold expression of F^2^ > 2sigma(F^2^) is used only for calculating R-factors(gt) etc. and is not relevant to the choice of reflections for refinement. R-factors based on F^2^ are statistically about twice as large as those based on F, and R- factors based on ALL data will be even larger.

### Fractional atomic coordinates and isotropic or equivalent isotropic displacement parameters (Å^2^)

**Table:**

	*x*	*y*	*z*	*U*_iso_*/*U*_eq_	
O1	1.03560 (11)	0.14592 (7)	1.07661 (12)	0.01241 (15)	
O2	1.29186 (11)	0.47147 (8)	1.22084 (12)	0.01554 (17)	
O3	0.81723 (11)	0.48152 (7)	0.83916 (11)	0.01375 (16)	
O4	0.58471 (11)	0.32057 (7)	0.97921 (12)	0.01402 (16)	
O5	0.41464 (11)	0.06573 (8)	0.55640 (12)	0.01527 (17)	
O6	0.94414 (10)	0.14952 (7)	0.66677 (12)	0.01358 (16)	
N1	1.16599 (12)	0.30914 (8)	1.13935 (13)	0.01118 (16)	
N2	1.05010 (13)	0.47550 (7)	1.03408 (14)	0.01113 (16)	
N3	0.50054 (11)	0.19796 (8)	0.76033 (12)	0.01090 (16)	
N4	0.67803 (12)	0.11318 (8)	0.60677 (13)	0.01124 (16)	
C1	1.03474 (13)	0.24831 (9)	1.05040 (14)	0.00980 (17)	
C2	1.17569 (14)	0.42182 (9)	1.13520 (15)	0.01120 (18)	
C3	0.92023 (13)	0.42115 (9)	0.93130 (15)	0.01017 (17)	
C4	0.91423 (13)	0.30599 (9)	0.93888 (15)	0.00967 (17)	
C5	0.77985 (13)	0.24525 (9)	0.83177 (14)	0.00979 (18)	
C6	0.62473 (13)	0.25855 (9)	0.86409 (14)	0.01016 (17)	
C7	0.52552 (13)	0.12277 (9)	0.63627 (15)	0.01075 (18)	
C8	0.80944 (13)	0.17050 (9)	0.70160 (15)	0.01024 (17)	
C9	1.29964 (15)	0.25137 (11)	1.24737 (16)	0.0154 (2)	
H9A	1.3982	0.2837	1.2299	0.023*	
H9B	1.2972	0.1752	1.2142	0.023*	
H9C	1.2912	0.2573	1.3689	0.023*	
C10	1.04993 (17)	0.59583 (9)	1.03645 (19)	0.0170 (2)	
H10A	1.1471	0.6218	1.1073	0.025*	
H10B	0.9608	0.6216	1.0848	0.025*	
H10C	1.0418	0.6231	0.9189	0.025*	
C11	0.34045 (15)	0.20432 (11)	0.80192 (18)	0.0174 (2)	
H11A	0.2627	0.1952	0.6964	0.026*	
H11B	0.3261	0.2747	0.8530	0.026*	
H11C	0.3274	0.1472	0.8837	0.026*	
C12	0.70481 (16)	0.04107 (12)	0.46407 (19)	0.0201 (2)	
H12A	0.6055	0.0087	0.4092	0.030*	
H12B	0.7785	−0.0159	0.5103	0.030*	
H12C	0.7480	0.0831	0.3789	0.030*	
N5	0.08583 (12)	0.04901 (9)	0.41174 (14)	0.01257 (17)	
H1N5	0.061 (3)	−0.020 (2)	0.410 (3)	0.027 (6)*	
H2N5	0.067 (3)	0.083 (2)	0.315 (3)	0.029 (5)*	
H3N5	0.195 (3)	0.0572 (17)	0.456 (3)	0.020 (5)*	
H4N5	0.035 (3)	0.0832 (18)	0.486 (3)	0.025 (5)*	
N6	0.56158 (13)	0.46002 (9)	0.56578 (14)	0.01434 (18)	
H1N6	0.566 (3)	0.4075 (16)	0.488 (3)	0.016 (4)*	
H2N6	0.465 (3)	0.4638 (17)	0.604 (3)	0.022 (5)*	
H3N6	0.568 (3)	0.5221 (19)	0.520 (3)	0.023 (5)*	
H4N6	0.641 (3)	0.4479 (18)	0.654 (3)	0.023 (5)*	
O1W	0.69194 (13)	0.30906 (8)	0.33669 (14)	0.01932 (18)	
H1W1	0.753 (3)	0.364 (2)	0.346 (3)	0.026 (5)*	
H2W1	0.667 (3)	0.297 (2)	0.236 (4)	0.033 (6)*	

### Atomic displacement parameters (Å^2^)

**Table:**

	*U*^11^	*U*^22^	*U*^33^	*U*^12^	*U*^13^	*U*^23^
O1	0.0146 (4)	0.0091 (3)	0.0126 (3)	0.0000 (3)	0.0001 (3)	0.0007 (3)
O2	0.0123 (4)	0.0164 (4)	0.0170 (4)	−0.0048 (3)	0.0002 (3)	−0.0031 (3)
O3	0.0144 (4)	0.0107 (3)	0.0147 (4)	0.0010 (3)	−0.0010 (3)	0.0005 (3)
O4	0.0141 (4)	0.0137 (4)	0.0147 (4)	0.0001 (3)	0.0039 (3)	−0.0042 (3)
O5	0.0098 (3)	0.0165 (4)	0.0179 (4)	−0.0020 (3)	−0.0017 (3)	−0.0043 (3)
O6	0.0096 (3)	0.0153 (4)	0.0158 (4)	0.0003 (3)	0.0024 (3)	−0.0037 (3)
N1	0.0091 (4)	0.0118 (4)	0.0117 (4)	−0.0009 (3)	−0.0005 (3)	−0.0001 (3)
N2	0.0107 (4)	0.0087 (3)	0.0133 (4)	−0.0016 (3)	0.0002 (3)	−0.0012 (3)
N3	0.0081 (4)	0.0126 (4)	0.0119 (4)	0.0000 (3)	0.0016 (3)	−0.0014 (3)
N4	0.0092 (4)	0.0112 (4)	0.0128 (4)	−0.0003 (3)	0.0009 (3)	−0.0041 (3)
C1	0.0086 (4)	0.0109 (4)	0.0098 (4)	−0.0006 (3)	0.0014 (3)	−0.0008 (3)
C2	0.0105 (4)	0.0122 (4)	0.0110 (4)	−0.0027 (4)	0.0025 (3)	−0.0010 (3)
C3	0.0098 (4)	0.0103 (4)	0.0102 (4)	−0.0009 (3)	0.0015 (3)	−0.0010 (3)
C4	0.0090 (4)	0.0090 (4)	0.0104 (4)	−0.0004 (3)	0.0001 (3)	−0.0015 (3)
C5	0.0097 (4)	0.0089 (4)	0.0102 (4)	−0.0004 (3)	0.0002 (3)	−0.0011 (3)
C6	0.0100 (4)	0.0092 (4)	0.0107 (4)	−0.0006 (3)	0.0004 (3)	0.0001 (3)
C7	0.0096 (4)	0.0095 (4)	0.0124 (4)	−0.0003 (3)	0.0001 (3)	−0.0002 (3)
C8	0.0101 (4)	0.0090 (4)	0.0109 (4)	−0.0009 (3)	0.0001 (3)	−0.0003 (3)
C9	0.0112 (4)	0.0172 (5)	0.0160 (5)	0.0002 (4)	−0.0023 (4)	0.0023 (4)
C10	0.0188 (5)	0.0099 (4)	0.0211 (5)	−0.0024 (4)	0.0004 (4)	−0.0014 (4)
C11	0.0102 (5)	0.0222 (6)	0.0204 (5)	−0.0007 (4)	0.0044 (4)	−0.0033 (4)
C12	0.0133 (5)	0.0243 (6)	0.0224 (6)	−0.0025 (4)	0.0027 (4)	−0.0145 (5)
N5	0.0120 (4)	0.0126 (4)	0.0130 (4)	−0.0014 (3)	0.0018 (3)	0.0006 (3)
N6	0.0141 (4)	0.0137 (4)	0.0147 (4)	−0.0007 (3)	0.0013 (3)	0.0008 (3)
O1W	0.0220 (5)	0.0170 (4)	0.0176 (4)	−0.0044 (3)	0.0001 (3)	−0.0003 (3)

### Geometric parameters (Å, °)

**Table:**

O1—C1	1.2611 (14)	C9—H9A	0.9600
O2—C2	1.2433 (14)	C9—H9B	0.9600
O3—C3	1.2611 (14)	C9—H9C	0.9600
O4—C6	1.2620 (14)	C10—H10A	0.9600
O5—C7	1.2427 (13)	C10—H10B	0.9600
O6—C8	1.2544 (14)	C10—H10C	0.9600
N1—C2	1.3733 (15)	C11—H11A	0.9600
N1—C1	1.4117 (14)	C11—H11B	0.9600
N1—C9	1.4642 (15)	C11—H11C	0.9600
N2—C2	1.3710 (15)	C12—H12A	0.9600
N2—C3	1.4052 (15)	C12—H12B	0.9600
N2—C10	1.4631 (14)	C12—H12C	0.9600
N3—C7	1.3713 (15)	N5—H1N5	0.86 (2)
N3—C6	1.4139 (14)	N5—H2N5	0.85 (3)
N3—C11	1.4623 (15)	N5—H3N5	0.94 (2)
N4—C7	1.3672 (15)	N5—H4N5	0.89 (2)
N4—C8	1.4072 (14)	N6—H1N6	0.88 (2)
N4—C12	1.4616 (16)	N6—H2N6	0.93 (2)
C1—C4	1.4033 (15)	N6—H3N6	0.84 (2)
C3—C4	1.4028 (15)	N6—H4N6	0.88 (2)
C4—C5	1.4830 (15)	O1W—H1W1	0.84 (3)
C5—C6	1.4013 (15)	O1W—H2W1	0.79 (3)
C5—C8	1.4143 (15)		
			
C2—N1—C1	123.86 (10)	N1—C9—H9B	109.5
C2—N1—C9	116.61 (10)	H9A—C9—H9B	109.5
C1—N1—C9	119.51 (10)	N1—C9—H9C	109.5
C2—N2—C3	123.53 (9)	H9A—C9—H9C	109.5
C2—N2—C10	118.11 (10)	H9B—C9—H9C	109.5
C3—N2—C10	118.34 (10)	N2—C10—H10A	109.5
C7—N3—C6	123.30 (9)	N2—C10—H10B	109.5
C7—N3—C11	117.50 (10)	H10A—C10—H10B	109.5
C6—N3—C11	118.70 (9)	N2—C10—H10C	109.5
C7—N4—C8	124.21 (9)	H10A—C10—H10C	109.5
C7—N4—C12	117.63 (10)	H10B—C10—H10C	109.5
C8—N4—C12	118.14 (10)	N3—C11—H11A	109.5
O1—C1—C4	125.05 (10)	N3—C11—H11B	109.5
O1—C1—N1	117.25 (10)	H11A—C11—H11B	109.5
C4—C1—N1	117.69 (9)	N3—C11—H11C	109.5
O2—C2—N2	122.47 (10)	H11A—C11—H11C	109.5
O2—C2—N1	121.16 (11)	H11B—C11—H11C	109.5
N2—C2—N1	116.36 (10)	N4—C12—H12A	109.5
O3—C3—C4	125.29 (10)	N4—C12—H12B	109.5
O3—C3—N2	116.25 (10)	H12A—C12—H12B	109.5
C4—C3—N2	118.46 (10)	N4—C12—H12C	109.5
C3—C4—C1	119.76 (10)	H12A—C12—H12C	109.5
C3—C4—C5	120.26 (9)	H12B—C12—H12C	109.5
C1—C4—C5	119.97 (9)	H1N5—N5—H2N5	117 (2)
C6—C5—C8	120.00 (9)	H1N5—N5—H3N5	109.6 (19)
C6—C5—C4	120.09 (9)	H2N5—N5—H3N5	107 (2)
C8—C5—C4	119.86 (10)	H1N5—N5—H4N5	108 (2)
O4—C6—C5	125.63 (10)	H2N5—N5—H4N5	108 (2)
O4—C6—N3	116.18 (10)	H3N5—N5—H4N5	107 (2)
C5—C6—N3	118.19 (9)	H1N6—N6—H2N6	113.9 (19)
O5—C7—N4	122.03 (11)	H1N6—N6—H3N6	110.3 (19)
O5—C7—N3	121.25 (10)	H2N6—N6—H3N6	103 (2)
N4—C7—N3	116.71 (10)	H1N6—N6—H4N6	106.3 (19)
O6—C8—N4	117.44 (10)	H2N6—N6—H4N6	111 (2)
O6—C8—C5	125.09 (10)	H3N6—N6—H4N6	112 (2)
N4—C8—C5	117.44 (10)	H1W1—O1W—H2W1	106 (3)
N1—C9—H9A	109.5		
			
C2—N1—C1—O1	−174.49 (10)	C3—C4—C5—C8	117.68 (12)
C9—N1—C1—O1	3.76 (15)	C1—C4—C5—C8	−63.31 (14)
C2—N1—C1—C4	5.98 (15)	C8—C5—C6—O4	178.22 (11)
C9—N1—C1—C4	−175.76 (10)	C4—C5—C6—O4	0.76 (17)
C3—N2—C2—O2	177.67 (11)	C8—C5—C6—N3	−1.73 (15)
C10—N2—C2—O2	−3.67 (17)	C4—C5—C6—N3	−179.19 (9)
C3—N2—C2—N1	−3.18 (16)	C7—N3—C6—O4	−176.44 (10)
C10—N2—C2—N1	175.47 (10)	C11—N3—C6—O4	−4.80 (15)
C1—N1—C2—O2	177.56 (10)	C7—N3—C6—C5	3.52 (15)
C9—N1—C2—O2	−0.74 (16)	C11—N3—C6—C5	175.16 (10)
C1—N1—C2—N2	−1.60 (16)	C8—N4—C7—O5	−175.66 (11)
C9—N1—C2—N2	−179.90 (10)	C12—N4—C7—O5	6.19 (17)
C2—N2—C3—O3	−176.92 (10)	C8—N4—C7—N3	3.66 (16)
C10—N2—C3—O3	4.43 (16)	C12—N4—C7—N3	−174.49 (11)
C2—N2—C3—C4	3.31 (17)	C6—N3—C7—O5	174.96 (10)
C10—N2—C3—C4	−175.34 (10)	C11—N3—C7—O5	3.22 (16)
O3—C3—C4—C1	−178.39 (11)	C6—N3—C7—N4	−4.37 (15)
N2—C3—C4—C1	1.36 (16)	C11—N3—C7—N4	−176.11 (10)
O3—C3—C4—C5	0.63 (18)	C7—N4—C8—O6	176.38 (10)
N2—C3—C4—C5	−179.62 (9)	C12—N4—C8—O6	−5.48 (16)
O1—C1—C4—C3	174.85 (11)	C7—N4—C8—C5	−2.08 (16)
N1—C1—C4—C3	−5.66 (15)	C12—N4—C8—C5	176.06 (11)
O1—C1—C4—C5	−4.17 (17)	C6—C5—C8—O6	−177.28 (11)
N1—C1—C4—C5	175.32 (9)	C4—C5—C8—O6	0.18 (17)
C3—C4—C5—C6	−64.86 (14)	C6—C5—C8—N4	1.05 (15)
C1—C4—C5—C6	114.15 (12)	C4—C5—C8—N4	178.51 (10)

### Hydrogen-bond geometry (Å, °)

**Table:**

*D*—H···*A*	*D*—H	H···*A*	*D*···*A*	*D*—H···*A*
N5—H1N5···O1^i^	0.87 (2)	2.04 (2)	2.7629 (14)	141 (2)
N5—H1N5···O6^i^	0.87 (2)	2.52 (2)	3.1697 (14)	132.9 (19)
N5—H2N5···O1^ii^	0.85 (2)	1.97 (2)	2.8116 (14)	173 (2)
N5—H3N5···O5	0.94 (3)	1.90 (3)	2.8312 (14)	176.1 (19)
N5—H4N5···O6^iii^	0.89 (2)	1.90 (2)	2.7819 (14)	173 (2)
N6—H1N6···O1W	0.88 (2)	2.10 (2)	2.9126 (15)	152 (2)
N6—H2N6···O2^iv^	0.93 (3)	2.03 (2)	2.9074 (15)	157.8 (19)
N6—H3N6···O4^v^	0.84 (2)	1.95 (2)	2.7665 (14)	164 (2)
N6—H4N6···O3	0.88 (2)	1.92 (2)	2.7597 (14)	158 (2)
O1W—H1W1···O3^v^	0.84 (3)	1.96 (2)	2.7602 (13)	158 (2)
O1W—H2W1···O4^vi^	0.78 (3)	2.01 (3)	2.7563 (14)	161 (3)
C9—H9A···O1W^vii^	0.96	2.51	3.3658 (17)	148
C10—H10C···Cg1^v^	0.96	2.96	3.8822 (15)	162

Symmetry codes: (i) *x*−1, −*y*, *z*−1/2; (ii) *x*−1, *y*, *z*−1; (iii) *x*−1, *y*, *z*; (iv) *x*−1, −*y*+1, *z*−1/2; (v) *x*, −*y*+1, *z*−1/2; (vi) *x*, *y*, *z*−1; (vii) *x*+1, *y*, *z*+1.
